# Screening of problems related to alcohol, tobacco and cannabis in primary care settings: a comparative study between Brazil and Portugal

**DOI:** 10.1186/1940-0640-10-S2-P2

**Published:** 2015-09-24

**Authors:** Angela Abreu, Rafael T Jomar, Rafaela Costa, Rachel FS Figueiro, Maria Helena N Silva, Pedro Parreira, Teresa Barroso

**Affiliations:** 1 Public Health Nursing, Federal University of Rio de Janeiro, Brazil; 2 State University of Rio de Janeiro, Brazil; 3 Federal University of Rio de Janeiro, Brazil; 4 Coimbra Nursing School, Portugal

## Background

The consumption of psychoactive substances is a global health problem.

The objectives are to identify the sample socio-demographic profile and use pattern of alcohol, tobacco and cannabis throughout life and in the last three months. Also, to discuss the application of Brief Intervention procedures for problems related to psychoactive substances in patients assisted at the primary health care, in Brazil (Rio de Janeiro) and Portugal (Coimbra).

## Material and methods

The sample included 1700 individuals (Brazil, n=1489; Portugal, n=211). All participants filled the instrument Alcohol, Smoking and Substance Involvement Screening Test. Analyses were performed (Student's t-test, ANOVA and Pearson's correlations) using the Statistical Package Social Science (SPSS) version 22.0. A level of statistical significance of 0.05 was established.

## Results

It was observed in both countries higher prevalence in attendance of female participants, married, income between 1 and 2 minimum wage (in Brazil). Considering lifelong use of substances, the Brazilian sample showed 45.5% of tobacco use, 67.6% of use of alcoholic beverages and 8.6% of cannabis use. In Portugal, tobacco (59.3%), alcoholic beverages (88.1%) and cannabis (13.5%). Considering the daily frequency of use in the last three months in Brazil: tobacco (14.7%), alcoholic beverages (2.8%), cannabis (0.7%); and in Portugal: tobacco (22.7%), alcohol (32.7%) and cannabis (0.5%). Individuals classified as “moderate risk” were selected to receive Brief Intervention: in Brazil, tobacco use (score 4-26) 16.6%, alcoholic beverages (score 11-26) 8.8%, cannabis 1.5%; Portugal tobacco use (32.0%), use of alcoholic beverages (19.3%) and cannabis use (12.7%).

## Conclusions

It was observed the use of psychoactive substances both countries and the importance of the primary health care in the early detection of health problems associated to the use of those substances. The scenario is responsible for health promoting/protection.
